# Supplementary material for: Chromothripsis during telomere crisis is independent of NHEJ, and consistent with a replicative origin
Source: Genome Res. 2019 May;29(5):737–49. doi: 10.1101/gr.240705.118 (PMC6499312; doi:10.1101/gr.240705.118)
Supplement: Supplemental Material [file supp_gr.240705.118_Supplemental_file_1.zip › contigs/annotated_contigs/DB41/contig.2.DB41_length_265_mean_cov_1.69811320755.docx]

**DB41_length_265_mean_cov_1.69811320755**

```
AGATGGCAAGGGCAAGGGAGCTGAGGCTGAACAGTCTTGATCTCTGAAACAGATGGTGTGCACGTGTGTGTGTGCATGTTTGTGTAAGT
 >chr17:75869156-75869378 - E=8e-120 p=0e+00
GTGTGCATGTTTGTGTAAGTGTGTGCATATGTGCGTGTGCGTGTTTGTGTGCGCATGTGTGTGCATGTGTTTGTGTCTGCATGTGTGTT

TGTGTGTGCGTGTGTGCGTGTGGGTGCTTGTGTGCGTGTGCGT|G|GGTGTGTGTGTGTTGGGGTGTGTTTGTGTGTG|GGTGGGTGTG
                                            >chr14:100633657-100633690 - E=1e-05
G
```
